# Continuous-Flow Stable Sulfur Isotope Analysis of Organic and Inorganic Compounds by EA-MC-ICPMS

**DOI:** 10.1021/acs.analchem.4c00439

**Published:** 2024-05-13

**Authors:** Axel Horst, Matthias Gehre, Marcus Fahle, Steffen Kümmel

**Affiliations:** † Department Technical Biogeochemistry, Helmholtz Centre for Environmental Research−UFZ, Permoserstr. 15, 04318 Leipzig, Germany; ‡ Research and Development Centre for Post-Mining Areas, Federal Institute for Geosciences and Natural Resources (BGR), Gaglower Str. 17-18, 03048 Cottbus, Germany

## Abstract

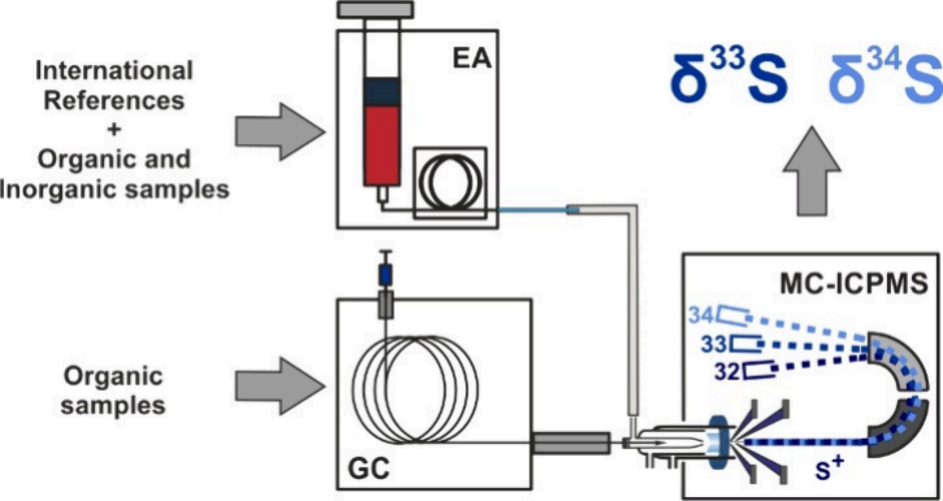

Elemental analysis (EA) coupled to isotope ratio mass spectrometry (IRMS) is a well-established method to derive stable isotope ratios of sulfur (^34^S/^32^S). Conversion of sulfur to SO_2_ by EA and measurement of SO_2_ isotopologues by IRMS represents the simplest and most versatile method to accomplish isotope measurement of sulfur even in bulk samples. Yet, interferences by oxygen isotopes in SO_2_ often impair the precision and trueness of measured results. In the current study, we coupled EA to multicollector inductively coupled plasma mass spectrometry (MC-ICPMS) to establish a method that avoids such interferences due to direct measurement of S^+^ ions. In addition, measurement of the ^33^S/^32^S isotope ratios is possible, thus representing the first bulk method that is suitable to study mass-independent isotope fractionation (MIF). Analytical precision (σ) of available Ag_2_S and BaSO_4_ reference materials (RMs) was, on average, 0.2 mUr for *δ*^33^S and *δ*^34^S, never exceeding 0.3 mUr within this study (1 mUr = 1‰ = 0.001). Measured *δ*^34^S values of reference materials agreed within ±0.2 mUr of officially reported values. Measurement of wood samples yielded good precision (0.2 mUr) for sulfur amounts as low as 3.5 μg, but precision deteriorated for samples at lower sulfur contents due to poor peak shape. Finally, we explored cross-calibration of organic liquids separated via gas chromatography (GC) against solid RMs combusted via EA that avoids challenging offline conversion of RMs. Results indicate good precision (≤0.08 mUr) and acceptable trueness (≤0.34 mUr) for determination of *δ*^34^S, demonstrating the future potential of such an approach.

## Introduction

Sulfur is an essential element for all life on earth. Present mainly as organosulfur compounds or metal sulfides, sulfur is widely distributed in virtually all reservoirs.^1^ Hence, stable sulfur isotopes have been analyzed in sediments and rocks,^2,3^ meteorites,^4^ water,^5^ atmosphere,^6^ and aerosols,^7^ as well as plant and animal tissue,^8^ to obtain information about chemical reactions and processes of sulfur cycling within and between these reservoirs. Stable isotope analysis of sulfur is considered challenging compared to isotope analysis of other traditional light elements (H, C, N, and O).^9^ These challenges are mainly linked to the measuring gas SO_2_ (sulfur dioxide) used in traditional offline^10^ and online methods,^11^ which reacts with surfaces causing memory effects during offline preparation, for example.^12^ Measurement of SO_2_ for sulfur isotope ratios further requires correction for variations in oxygen isotopes, which is not as well implemented as correction for oxygen in CO_2_.^13^ Despite these challenges, online conversion of sulfur to SO_2_ via elemental analysis (EA) and subsequent measurement of ^34^S/^32^S isotope ratios via isotope ratio mass spectrometry (IRMS) belongs to the most versatile and rapid methods and hence is widely used in (bio)geosciences, hydrology, and soil sciences.

As an alternative to SO_2_, sulfur hexafluoride (SF_6_) has been used as a measuring gas in IRMS methods.^4^ SF_6_ is suitable to also analyze the rare isotopes of sulfur (^33^S and ^36^S), which opens up the opportunity to investigate processes that cause mass-independent isotope fractionation (MIF) such as photochemical reactions.^14,15^ Even though sulfur isotope measurements using SF_6_ are more accurate than those obtained from SO_2_ measurements,^16,17^ the laborious extraction technique involving fluorination is less appropriate for measurement of larger sample numbers.^9^

Nontraditional methods for sulfur isotope analysis comprise secondary ion mass spectrometry (SIMS),^18^ thermal ionization mass spectrometry (TIMS),^19^ and multicollector inductively coupled plasma mass spectrometry (MC-ICPMS)^20,21^ that were developed to meet the various demands in each field. Particularly, MC-ICPMS in combination with laser ablation for solids^20^ and spray chamber/desolvation for liquids^21^ has become a more common technique because it allows for measurement of much smaller sample amounts down to 5–40 nmol S with the benefit of also obtaining ^33^S/^32^S isotope ratios.^22^ Challenges arise from isobaric interferences, instrumental mass bias, and blank effects.^16^ Laser ablation measurements further require matrix-matched samples and reference materials to achieve acceptable accuracy.^20^

In the study presented here, we coupled elemental analysis (EA) with MC-ICPMS in order to investigate whether the advantages of both techniques are combinable. EA provides rapid and simple online conversion of organic and inorganic sulfur to SO_2_, including separation from other combustion products, and thus allows for direct measurement of natural samples omitting laborious preparation or offline conversion. The plasma of the MC-ICPMS decomposes sulfur to generate S^+^ ions and hence avoids oxygen interferences that affect results of conventional IRMS methods using SO_2_ as a measuring gas.^13^ The method was tested by using a set of international reference materials and wood samples to test its applicability for natural samples. Previously referenced organic material was used to explore the simultaneous use of gas chromatography (GC) and EA for cross-referencing GC-amenable compounds directly against solid reference material without conversion to SF_6_.

## Experimental Section

### Reference Materials and Measured Samples

Sulfur isotope reference materials (IAEA-S-1, IAEA-S-2, IAEA-S-3, IAEA-SO-5, IAEA-SO-6, NBS-127, and NBS-123) were provided by the International Atomic Energy Agency (IAEA) in Vienna, Austria. Additionally, the following chemicals were analyzed: Ag_2_SO_4_ (VEB Arzneimittelwerk Dresden, GDR), (NH_4_)_2_SO_4_ (Riedel de Haën AG, Germany), Na_2_S_2_O_8_ (Carl Roth GmbH, Germany), l-cysteine (C_3_H_7_NO_2_S), and Ag_2_S (Sigma-Aldrich, USA). The purity was above 97% (l-cysteine) and 99% (all other chemicals). The US Geological Survey (USGS), Reston, Virginia, provided a wood sample of Mexican ziricote (*Cordia dodecandra*, USGS55). The saxaul sample (*Haloxylon ammodendron*) was provided by the IAEA. Furthermore, previously isotopically characterized organics were analyzed:^23^ C_4_H_8_S (tetrahydrothiophene, THT), C_4_H_4_S (thiophene, THI), and C_4_H_10_S (diethyl sulfide, DES) purchased from Sigma-Aldrich, USA; C_2_H_6_S_2_ (dimethyl disulfide, DMDS) purchased from ABCR GmbH, Germany. Sulfur hexafluoride (SF_6_) was purchased from Linde AG, Germany.

### Preparation of Samples for Measurement

For EA measurements, solid samples were weighed, mixed with equal amounts of V_2_O_5_, and crimped in tin capsules. Liquid organic compounds were first diluted with hexane (1:10) and placed with a gastight syringe into tin capsules that contained 2 mg of Al_2_O_3_ (ComAid, LECO) to reduce evaporation. Liquid samples were prepared immediately prior to analysis, keeping the tin capsules closed with a pair of forceps, dropping the sample less than 5 s before actuation of the autosampler. For GC analyses, 250 mL septum bottles were flushed with N_2_ and crimp sealed with gray Wheaton stoppers. To prepare the gas mixture, 0.5 mL of gaseous SF_6_ or 1 μL of organic liquid was injected with a gastight syringe and equilibrated for 1 h before measurement.

### Instrumental Setup and Data Acquisition

**Figure 1:**
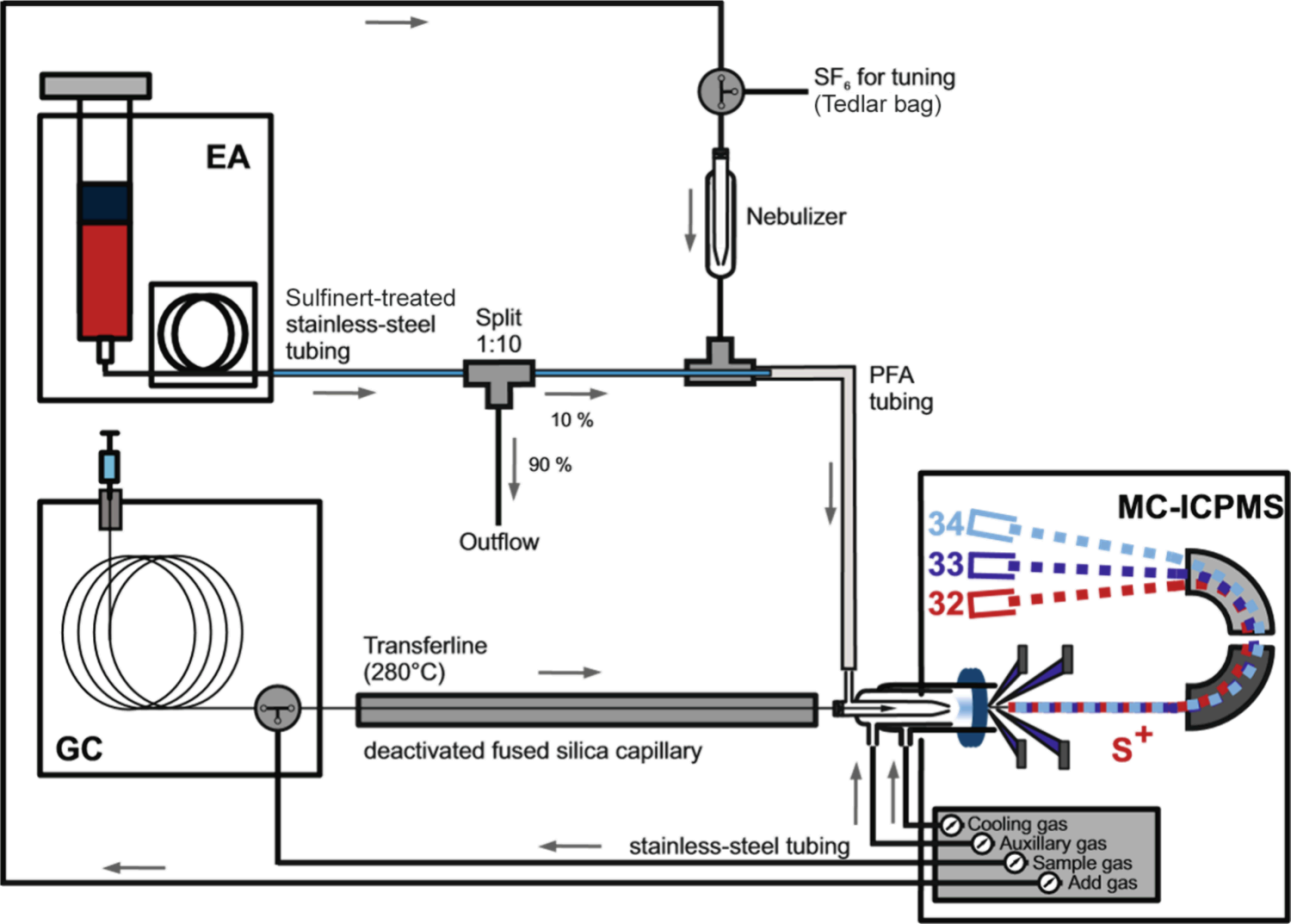
Instrumental setup.

Conversion of sulfur to SO_2_ in the various samples was accomplished with an elemental analyzer (EuroVector 3000, Milano, Italy) equipped with a standard reactor packed with tungsten oxide, quartz wool, and copper. The EA was operated with argon (flow rate 80 mL min^–1^) instead of helium as carrier gas, because the better separation efficiency of helium is not needed for EA applications. Since argon is also used to maintain and cool the plasma in the ICP, usage of this more available gas appeared reasonable. Conversion of sulfur in the samples was achieved via flash combustion with an oxygen pulse of 15 mL. A quartz insert was used to retain combustion residuals. Before entering the column (70 °C) for separation (0.8 m × 6.35 mm PTFE packed with Porapack 50–80 mesh, HEKAtech Analysenservice GmbH, Germany), the produced gases passed through a water trap filled with P_2_O_5_ as the drying agent. The connection to the torch of the ICP was established via a custom-made interface (Figure 1). After leaving the reactor, 10% of the gases were directed via Sulfinert-tubing (Restek, Germany) and a heated metal capillary into a 6.35 mm (1/4 in.) perfluoroalkyl tube (PFA). Here, the gases were diluted by argon (500 mL min^–1^ add gas flow) and rapidly transported to the ICP torch for ionization. Apart from the EA, we also connected a gas chromatographic system (GC, Trace 1310, Thermo Fisher Scientific, Germany) via a heated transferline (AE2080, Aquitaine Electronique, France) to the torch of the ICP for direct introduction of organic compounds.^23^ Detailed parameters are listed in Table S1 in the Supporting Information or can be found in Kümmel et al.^23^

Tuning and optimization of the MC-ICPMS was achieved by introducing an SF_6_/Ar gas mixture (1% SF_6_) via a nebulizer incorporated into the add gas flow line (see Figure 1). Parameters such as torch position, ion extraction lenses, and focus were checked daily and adjusted for maximum sensitivity. The sample gas flow in combination with the add gas flow was optimized for a maximum signal-to-noise ratio instead of maximum sensitivity to keep isobaric interferences as low as possible.^23,24^ Combined with dry plasma conditions, potential isobaric interferences (e.g., ^16^O^16^O^+^, ^32^SH^+^) were negligible and the MC-ICPMS system was operated in low mass resolution mode (*m*/Δ*m* ≈ 300), as demonstrated previously.^23,24^ Dry plasma means that only the carrier gas argon containing SO_2_ is introduced into the plasma, which contrasts with conventional dry plasma applications using a desolvator, where liquids are still present as carriers of the analytes. Three Faraday detectors were positioned to simultaneously record the signals of mass 32, mass 33, and mass 34.

### Data Acquisition and Scale Normalization

The computer of the MC-ICPMS uses Multi Collector Software, Version 3.2, to record the signals with a dwell time of 0.131 s and to export them to ASCII files. Isotopic ratios were calculated by integrating the areas of the transient signals (chromatographic peaks) using R (version 4.3.1)^25^ and the zoo package.^26^ The start and the end of the peaks were defined by slopes of +0.004 and −0.004, respectively, calculated for 11 consecutive points. The background was determined by the median of the 10 points preceding the peak. The script also provides the possibility to calculate isotopic ratios via the regression method, which showed benefits in terms of precision for GC-MC-ICPMS analyses of organics.^23,24^ For EA analysis, in contrast, peak integration showed more robust results in terms of trueness, and hence this isotope ratio calculation method was preferred. The script (file R_script) was added to the Supporting Information as well as a documentation of the script (SI1). Calculated isotopic ratios of each run were referenced against Ag_2_SO_4_, which was analyzed between sample runs containing up to 5 analyses. Isotopic ratios were expressed in *δ* notation
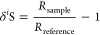
1where *i* indicates 33 or 34 and *R*_sample_ and *R*_reference_ are the ^34^S/^32^S or ^33^S/^32^S isotope ratios of the sample and the reference, respectively. *δ* values may be expressed in Ur (urey) or per mil (‰) according to IUPAC guidelines (1 mUr (milliurey) = 0.001 = 1‰).^27,28^ In this study, the *δ* values are expressed in Ur.

Raw *δ* values were normalized to the VCDT scale (Vienna-Canyon Diablo Troilite) using the sulfur isotope reference materials IAEA-S-1, IAEA-S-2, and IAEA-S-3. IAEA-S-1 is the primary reference material for sulfur with a recommended δ^34^S value of −0.3 mUr on the VCDT scale.^29^ We conducted linear two-point normalization of the raw *δ* values using the updated values of IAEA-S-2 and IAEA-S-3 reported by Mann and co-workers.^16^ There are no official consensus values for *δ*^33^S. Hence, we used the values published by Ding et al. for normalization of *δ*^33^S values.^30^

Coupling EA to MC-ICPMS provides the opportunity to study mass-independent isotope effects expressed as Δ^33^S in Ur, which is the deviation of *δ*^33^S from theoretic *δ*^33^S expected for mass-dependent fractionation:^4,14,31^
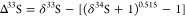
2Δ^33^S values were calculated for all reference materials and previously uncharacterized chemicals in order to get an impression of the utility of this bulk method to also study mass-independent isotope effects.

## Results and Discussion

### Conversion Efficiency, Blank Effects, and Memory

**Figure 2:**
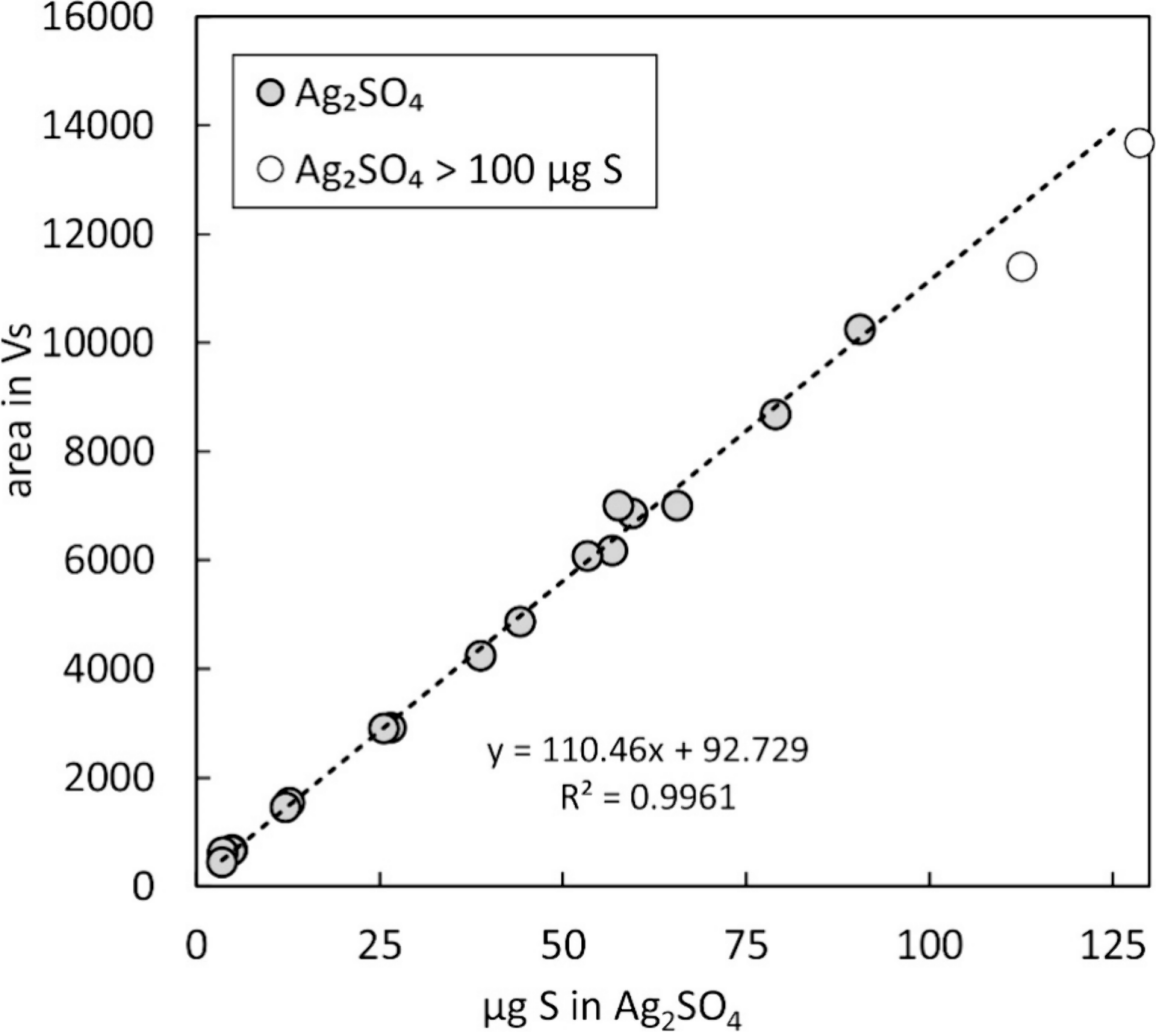
Linear relation between the amount of sulfur in Ag_2_SO_4_ samples and area of the recorded S^+^ peaks (mass 32) indicating consistent conversion of Ag_2_SO_4_ to SO_2_ and further to S^+^ in the plasma over a relatively large range of 3–90 μg of sulfur. Above 100 μg S conversion efficiency decreased. The regression line indicates the best fit of data designated by the gray circles.

**Figure 3:**
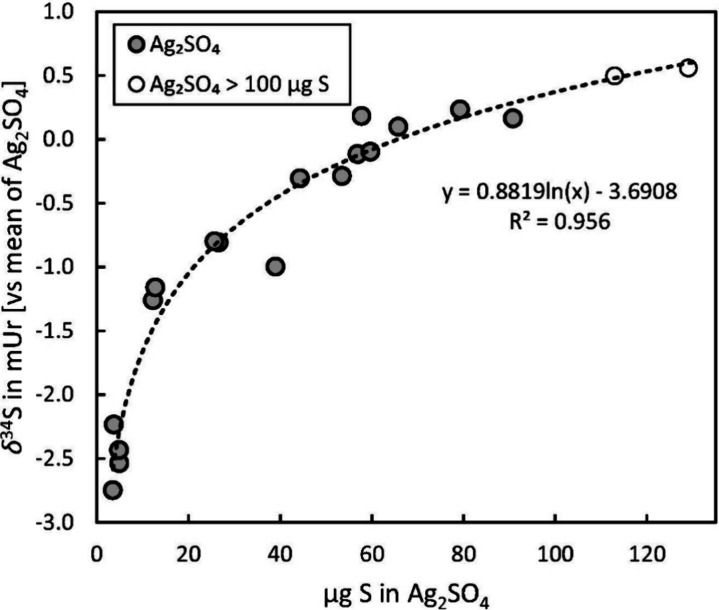
Logarithmic relationship between *δ*^34^S and the amount of sulfur. The equation represents the best fit for Ag_2_SO_4_ samples containing less than 90 μg S. Values are referenced against the mean *δ*^34^S of eight samples containing between 45 and 90 μg S. Samples above 100 μg S still fit this line, indicating predictable isotopic ratios despite lowered SO_2_ yields as indicated in Figure 2.

After the EA-MC-ICPMS system was carefully set up and tuned, several test series were run in order to evaluate the conversion of sulfur to SO_2_ in the EA and further to S^+^ in the plasma. Conversion behavior was evaluated with samples of Ag_2_SO_4_ containing 3–128 μg of sulfur. Ag_2_SO_4_ was chosen because of its fine grain size and high molar-mass-to-sulfur ratio, which makes it more convenient to prepare samples with low sulfur contents. The 17 Ag_2_SO_4_ samples analyzed in random order indicated a linear relationship between the amount of sulfur in the sample, the produced SO_2_, and the finally recorded S^+^ signals, suggesting a consistent and near-quantitative conversion in the reactor and ionization by the ICP (Figure 2). Samples larger than 100 μg of S showed a slightly lower signal area (5–10%) compared with the expected signal areas indicated by the linear trendline, which might be due to lower SO_2_ yields. These yields, however, did not measurably affect isotopic ratios, as indicated in Figure 3.

Blank effects during MC-ICPMS analyses might become a limiting factor especially for accurate low-level sulfur isotope measurements.^32^ In order to test whether trace amounts of sulfur were present in added compounds and capsules, we combusted empty tin capsules but also capsules filled with 100 μg of V_2_O_5_ or 2 mg of ComAid. In all instances, no sulfur signal was detected, and hence, no interference by additional sulfur is expected for the concentration range tested in this study. It is important to note at this point that the method was not set up for maximum sensitivity. Sulfur amounts in all samples were larger than 100 nmol. Previous studies identifying blank effects optimized their MC-ICPMS methods for maximum sensitivity and measured sulfur isotope ratios in the lower nanomol range of sulfur, and hence effects of blank were observed.^22,32^

Memory and carryover effects were investigated because SO_2_ is highly polar and shows a tendency to stick to surfaces.^9^ To avoid these effects as much as possible, at least 7 min passed between consecutive analyses in order to purge the remaining SO_2_ from the system. In this context, careful adjustment of the reactor temperature appeared imperative. At temperatures below 1020 °C peak tailing became more prominent probably due to retention of SO_2_ on Cu in the lower and cooler parts of the reactor. Temperatures above 1050 °C lead to melting of the copper in the upper parts of the reactor, even though 6 cm of WO_3_ was packed between the point of combustion and the copper packing. This might be related to argon, which was used as carrier gas and which possesses a slightly larger specific heat capacity (0.93 J dm^–3^ K) than helium (0.91 J dm^–3^ K) and thus heat produced during flash combustion may be transported to lower parts of the reactor, leading to a different temperature profile than expected for helium. Potential influence of memory and carryover was tested using IAEA reference materials. Four to five analyses were carried out for each Ag_2_S reference material with no other compounds or blank combustions interspersed. There was no detectable carryover between each set of samples, even though the difference in *δ*^34^S between IAEA-S-1 and IAEA-S-2 is +22.9 mUr. IAEA-S-2 and IAEA-S-3 differ by −55.1 mUr (Figure S1). This is also reflected in the standard deviation of *δ*^34^S, which was better than 0.2 mUr for each set of reference material (σ < 0.12 mUr for *δ*^33^S). These tests indicated that SO_2_ was not detectably retained in the EA system and the newly constructed interface and that memory and carryover may be considered negligible.

### Precision and Sample Size Dependence of Isotopic Ratios

Analytical precision is usually expressed as standard deviation (σ or sigma) and describes the closeness of agreement of a set of samples of the same material measured under near-identical conditions. Analyses of different organic and inorganic solid and liquid reagents resulted in standard deviations of at most 0.31 mUr for both *δ*^33^S and *δ*^34^S. On average, standard deviations of 0.1 mUr for *δ*^33^S and 0.15 mUr for *δ*^34^S were achieved for reference materials and previously uncharacterized solid materials (Table 1). This precision was robust and repeatable, as indicated by the results of the IAEA-Ag_2_S reference materials and Ag_2_SO_4_, which were measured throughout 4 different measurement campaigns. Overall standard deviations for Ag_2_S were smaller than 0.14 mUr (*n* = 12) for *δ*^33^S and *δ*^34^S, respectively. For Ag_2_SO_4_, standard deviations were 0.11 and 0.27 mUr (*n* = 12) for *δ*^33^S and *δ*^34^S, respectively. These results indicate that precision of our method for determination of ^34^S/^32^S isotope ratios is on a par with reported EA-IRMS methods^8,11^ with the benefit of additionally obtaining ^33^S/^32^S isotope ratios.

**Table 1:** Stable Sulfur Isotopic Composition (*δ*^33^S, *δ*^34^S) of International Reference Materials and Organic and Inorganic Compounds^1^

		reported values				measured values						deviation		MIF	
		δ^33^S	σ	*δ*^34^S	σ	*δ*^33^S	σ	*n*	*δ*^34^S	σ	*n*	*δ*^33^S	*δ*^34^S	Δ^33^S	σ
EA-MC-ICPMS Measurements															
IAEA-S-1	Ag_2_S	–0.05^a^	0.35	–0.30^a^		+0.16	0.07	12	–0.19	0.13	12	+0.21	–0.11	+0.12	0.09
IAEA-S-2	Ag_2_S	+11.57^a*^	0.22	+22.62^*^	0.16	+11.57	0.12	13	+22.62	0.14	13			–0.08	0.12
IAEA-S-3	Ag_2_S	–16.61^a*^	0.31	–32.49^b*^	0.16	–16.61	0.10	12	–32.49	0.14	12			+0.08	0.13
NBS-123	sphalerite			+17.44^c^	0.10	+9.07	0.05	3	+17.56	0.05	3		+0.12	–0.05	0.02
NBS-127	BaSO_4_			+21.12^c^	0.22	+11.03	0.06	3	+21.15	0.11	3		+0.03	+0.09	0.01
IAEA-S0–5	BaSO_4_			+0.49^c^	0.11	+0.79	0.03	3	+1.02	0.16	3		+0.53	+0.12	0.10
IAEA-S0-6	BaSO_4_			–34.05^c^	0.08	–17.46	0.29	3	–34.28	0.31	3		+0.23	+0.13	0.13
															
	Ag_2_SO_4_					+4.05	0.11	12	+7.71	0.27	12			0.00	0.13
	Ag_2_S					+10.39	0.04	3	+20.13	0.06	3			–0.02	0.04
	l-cysteine					+0.21	0.07	3	+0.07	0.14	3			–0.05	0.00
	(NH_4_)_2_SO_4_					+3.08	0.07	3	+5.36	0.04	3			+0.11	0.06
	Na_2_S_2_O_8_					+1.01	0.18	3	+1.49	0.28	3			+0.02	0.05
															
	THI	–1.74^d^	0.18	–3.35^d^	0.07	–2.13	0.15	3	–3.89	0.23	3	–0.39	–0.54	–0.11	0.05
	THT	+4.13^d^	0.17	+7.82^d^	0.15	+3.77	0.07	3	+7.59	0.15	3	–0.36	–0.23	–0.07	0.03
	DMDS	+8.60^d^	0.17	+16.97^d^	0.07	+8.11	0.08	3	+16.37	0.09	3	–0.49	–0.60	–0.20	0.05
	DES	+6.36^d^	0.20	+12.54^d^	0.17	+6.05	0.04	3	+12.21	0.13	3	–0.31	–0.33	–0.15	0.07
															
	saxaul								+4.54	0.16	3				
USGS55	Mexican ziricote								+10.72	0.93	3				
GC-MC-ICPMS Measurements Referenced against EA-MC-ICPMS Measurements															
	THI	–1.74	0.18	–3.35	0.07	–2.13	0.10	5	–3.69	0.06	5	–0.39	–0.34	–0.14	0.12
	THT	+4.13	0.17	+7.82	0.15	+3.56	0.13	5	+7.48	0.08	5	–0.57	–0.34	–0.26	0.15
	DMDS	+8.60	0.17	+16.97	0.07	+8.23	0.15	5	+16.96	0.05	5	–0.37	–0.01	–0.31	0.16
	DES	+6.36	0.20	+12.54	0.17	+6.17	0.05	3	+12.53	0.03	3	–0.19	–0.01	–0.12	0.05

1 Measured *δ*^34^S values were referenced against VCDT by using IAEA Ag_2_S reference materials and applying two-point normalization based on updated *δ*^34^S values of Mann et al.^16^ Published values of Ding et al.^30^ for IAEA Ag_2_S reference materials were used for normalization of *δ*^33^S values. All *δ* values are expressed in milliurey (1 mUr = 0.001 Ur = 1‰). The number of replicates is given by *n*. Deviation indicates the difference from reported values. Δ^33^S was determined using eq 2.

Reference materials used as anchors for two-point normalization are marked with an asterisk (*). ^a^ Mann et al.^16^^b^ Ding et al ^30^

c Brand et al.^28^

d Kümmel et al.^23^

The dependence of the measured isotopic ratio on the sample size (amount of sulfur) is also termed linearity, irrespective of the actual nature of relationship (linear or nonlinear). In our study, *δ*^34^S and the sample amount of Ag_2_SO_4_ showed a distinct nonlinear relationship as shown in Figure 3. Such sample size dependence of the isotopic ratio was also reported for sulfur organic compounds introduced into the plasma via a gas chromatographic system.^23^ This observation is probably related to so-called space charge effects in the plasma causing isotopic fractionation which might change with increasing amounts of analyte.^33^ Irrespective of the reasons, such changes must be accounted for when linking measured isotopic ratios of samples to the VCDT scale. The trendline in Figure 3 shows a relatively steep increase for sulfur amounts of 3–30 μg S, flattening out above 50 μg S. Between 45 and 90 μg S, variations due to sample size dependence become small, which is indicated by a standard deviation of 0.14 and 0.21 mUr for *δ*^33^S and *δ*^34^S (*n* = 8), respectively. Hence, measurements in this range are sufficiently precise for most applications. Similar precision over the entire range (3–128 μg) may be achieved by correcting measurements according to the logarithmic trendline shown in Figure 3. Even though such a procedure is convenient in day-to-day lab operation, we recommend matching sulfur amounts in samples and reference materials with a maximum variation of ±20%. Ionization in the plasma is highly susceptible to flow changes, and thus it cannot be completely excluded that the slope of the curve in Figure 3 changes on different measurement days due to slight variations of sample gas flow, add-gas flow, or carrier gas flow. Consequently, all measurements involving referencing to the VCDT scale were carried out by matching sulfur amounts in samples and reference materials (maximum ±20%) to avoid any effects due to sample size dependence.

### Measurement of Solids, Liquids, and Wood

A set of reference materials provided by the IAEA was analyzed in order to verify the newly developed method (Table 1). Isotopic ratios (^34^S/^33^S, ^33^S/^32^S) were measured for those reference materials as well as for some previously characterized organic liquids,^23^ unreferenced solids, and wood samples obtained from the USGS and IAEA and measured ratios were linked to VCDT. For *δ*^33^S no consensus values were available for most of these reference materials. For *δ*^34^S, all but one reference material agreed within 0.23 mUr with previously published values. IAEA-SO-5, a barium sulfate, showed a larger deviation of 0.53 mUr. IAEA-SO-6 and NBS-127 barium sulfates, in contrast, showed a good agreement with published values, and hence, this larger deviation from the consensus value is likely not related to differences in conversion between BaSO_4_ and IAEA-Ag_2_S reference materials, which were used to calibrate the measurements. When using the two BaSO_4_ reference materials IAEA-SO-6 and NBS-127 as anchors for two-point calibration, a value of +1.07 mUr is calculated for IAEA-SO-5, similar to +1.02 mUr obtained from calibration with the Ag_2_S reference material. The difference from the reported value of +0.49 mUr relative to VCDT might be related to the new methodology recording the isotopes of S^+^ ions instead of SO_2_^+^ or SO^+^ ions. Especially, results obtained from SO_2_ measurements are prone to interferences by oxygen isotopes, which might lead to erroneous *δ*^34^S values.^12,13^ The accepted value for NBS-127, for example, was previously corrected from +20.3 mUr to 21.1 mUr relative to VCDT after remeasurement as SF_6_.^12^ It is striking, in this respect, that IAEA-SO-5 yields a nearly correct value of +0.56 mUr if NBS-127 (together with IAEA-SO-6) is used as an anchor for two-point calibration assuming the formerly accepted value of +20.3 mUr. There is, however, no information on how IAEA-SO-5 was originally characterized, and we are not able to assess the true reasons for the observed offset. This example highlights, one more time, the importance of reporting isotopic values of used reference materials and the applied methods to provide information that is crucial to properly linking measured isotopic values to the currently accepted scale.

In addition to reference materials with previously published *δ*^34^S values, also some compounds with unknown isotopic values were measured including Ag_2_S, l-cysteine, (NH_4_)_2_SO_4_, and Na_2_S_2_O_8_. Values for *δ*^34^S ranged from +0.07 to +20.13 mUr and for *δ*^33^S from +0.21 to +10.39 mUr, representing enrichment of the heavier isotopes ^33^S and ^34^S compared to the zero-point of the VCDT scale (Table 1). Standard deviation ranged from 0.04 to 0.28 mUr and from 0.04 to 0.18 mUr for *δ*^34^S and *δ*^33^S values, respectively, indicating a generally robust precision for different kinds of solid materials.

Mass-independent isotope effects (Δ^33^S) were mainly calculated to obtain an impression of the precision of the EA-MC-ICPMS measurements to determine such effects. We cannot comment on the trueness of Δ^33^S values determined by EA-MC-ICPMS because respective reference materials with Δ^33^S values distinctly different from zero have only recently been introduced^34^ but were not available for the current study. There are reports of Δ^33^S values for IAEA-S-1 ranging from +0.014 to +0.11 mUr,^7,15,17,35^ agreeing well with +0.12 mUr found in the current study. Other solid compounds measured in the current study showed Δ^33^S values of −0.08 mUr (IAEA-S-2) to +0.13mUr (IAEA-SO-6). The standard deviation of those determinations reached up to 0.13 mUr, indicating that this EA-MC-ICPMS method shows a precision similar to that previously reported for organics measured via GC-MC-ICPMS.^23^ This is less precise than former published methods using offline SF_6_ conversion and dual inlet measurement, but in contrast to those methods, it allows for direct determination of sulfur isotopes in solid materials without the need for laborious preparation and separation techniques. The precision achieved may also be sufficient for various applications in isotope (bio-) geochemistry aiming at processes that cause MIF because the recommended threshold value for identification of a mass independent fractionation process is 0.20 mUr,^36^ which is resolvable with the presented method.

Apart from pure chemicals, the *δ*^34^S values of two wood samples were determined. Values of *δ*^33^S are not reported because the low sulfur content did not allow for a reliable determination. Sulfur contents in wood are generally low, with reported levels being in the range of about 50–400 mg kg^–1^ for spruce, oak, beech, pine, or poplar, for example.^37^ The two measured wood samples contained similar sulfur amounts of about 650 and 220 mg kg^–1^ for saxaul and ziricote, respectively. Triplicate measurements yielded *δ*^34^S values of +4.54 ± 0.16 mUr VCDT (saxaul) and +10.72 ± 0.93 mUr (ziricote), which are in the same range as published values for other wood species.^38,39^ Whereas analysis of saxaul showed good precision, results for ziricote indicated a lower precision. This lower precision is probably related to substantial peak broadening due to relatively large sample weights of 15 mg. Combustion of such large samples produces considerable amounts of CO_2_ and nitrogen species, causing substantial peak broadening during separation on the GC column of the EA. Signals could potentially be improved by applying more sophisticated methods such as cryotrapping SO_2_^40^ or using a dual-column GC-system for separation of CO_2_, N_2_, and SO_2_.^8^ Some more modern EA systems can achieve better separation and peak shape by trapping SO_2_ on a cold column and releasing it by rapidly heating the GC column. None of these techniques were available during the current study, and hence, stable sulfur isotope analysis of plant material such as wood will be limited to concentrations larger than 650 mg kg^–1^ when using the presented method.

Measurement of organic liquids via EA appeared more challenging in terms of trueness than the analysis of solids. Measured values showed a systematic negative offset of up to −0.6 mUr from previously published values (Table 1). Organic liquids were first diluted in hexane to be able to use a syringe to measure and draw up the small amounts of compound required for analysis. In a second step, liquids were placed in tin capsules containing an absorbent (ComAid) in order to reduce potential evaporation. Preliminary tests indicated that samples could be prepared only directly before analysis to minimize loss. Holding tin capsules crimped with forceps appeared to be essential. Despite these precautions, a shift of *δ*^34^S values was observed. Evaporation is generally known to produce small isotopic shifts in organic compounds for elements such as hydrogen, carbon, or chlorine.^41^ To reach a magnitude that is detectable, however, a relatively large amount has to evaporate because this process follows the Rayleigh type of isotopic fractionation.^9^ In addition, the observed shift would indicate an inverse sulfur isotope effect due to evaporation, for which there are no accounts in the literature. The small offset may also be explained with matrix differences and associated differences in the combustion of solid and liquid samples. Results of elemental analysis usually show good trueness even when comparing different sample materials, and matrix matching is not as mandatory as for other methods. The results of our study indicate, however, that referencing organic liquids directly against solid inorganic material using EA caused larger, for some applications, less acceptable, shifts of the isotopic composition. The procedure of preparing and analyzing tin capsules filled with organic liquids added additional factors of uncertainty that possibly impaired the reproducibility of such measurements.

### Exploring a GC/EA-MC-ICPMS Crossover Application

The setup presented in this study allows for a simultaneous functional connection of an EA system as well as a GC system to the torch of the ICP (Figure 1) and hence we hypothesized that crossover applications using both peripherals might be feasible. Specifically, we explored whether organic compounds directly injected and separated in a GC system could be referenced against solid materials converted by EA to SO_2_. Principally, such an approach is feasible because organics and SO_2_ decompose in the hot plasma to a multitude of ionic species of which only ^32^S^+^, ^33^S^+^, and ^34^S^+^ ions are recorded by the Faraday cups of the MC-ICPMS. Method-specific isotopic fractionation would have to be monitored and, if necessary, corrected for each peripheral.

We tested crossover referencing by separating and analyzing the four organic compounds directly via GC-MC-ICPMS and referenced those organics against IAEA standards S-1, S-2, and S-3 combusted and measured in the EA-MC-ICPMS system. Ag_2_SO_4_ was analyzed along with IAEA reference compounds via EA. SF_6_ was analyzed together with organic compounds via GC. Both Ag_2_SO_4_ and SF_6_ acted as internal working reference compounds within the respective methods. The offset between the assumed true values and measured values for Ag_2_SO_4_ and SF_6_ was used to correct for method differences. Results of crossover measurements indicate an overall higher accuracy than EA measurements of organic liquids. Especially for *δ*^34^S measurements, the standard deviation was consistently below 0.08 mUr, compared to 0.23 mUr with EA, and measured values agreed within 0.34 mUr with previously reported values, in contrast to differences of up to 0.6 mUr for EA determinations. For *δ*^33^S measurements via GC, precision and trueness were in the same range as those for EA measurements (Table 1). Δ^33^S values were slightly larger (up to 0.31 mUr) with standard deviations of up to 0.16 mUr compared to a maximum of 0.07 mUr for EA measurements. This lower accuracy might be related to the lower abundance of ^33^S, which makes ^33^S/^32^S ratio determination more susceptible to slight differences in peak shape and associated peak identification as well as baseline determination procedures for GC and EA measurements, respectively.

This exercise demonstrates that crossover referencing, in principle, is possible with satisfactory accuracy. Especially, *δ*^34^S determination of organic compounds separated on a GC works well when referenced against solid compounds converted to SO_2_ in an EA with both peripherals being connected to the same MC-ICPMS. For determination of Δ^33^S further fine-tuning will be required if calibration against solid reference materials with distinct Δ^33^S values such as S-MIF-1 and S-MIF-2 is planned.^34^

## Conclusions

The results presented in this study demonstrate that connecting an EA with an MC-ICPMS provides a valuable method that combines the simplicity of continuous flow SO_2_ conversion with interference-free and precise determination not only of *δ*^34^S but also of δ^33^S. It represents, to our knowledge, the first online method that is able to directly measure *δ*^33^S in bulk organic and inorganic samples, potentially allowing for determination of MIF in soil and plant samples, for example. The absence of oxygen interferences makes it a well-suited method for evaluation and determination of the sulfur isotope composition of solid reference material. Isotopic characterization of organic liquids and gases is usually accomplished by converting Ag_2_S reference materials to SF_6_ to create GC-amenable compounds. This challenging and laborious fluorination procedure may be avoidable in the future, as indicated by cross-calibration experiments. Organics injected in a GC may be directly referenced against Ag_2_S reference materials combusted in the EA with both peripherals connected to the same MC-ICPMS. Even though there is still room for improvement for *δ*^33^S measurements and consequently Δ^33^S analysis, cross referencing already provides satisfying results for determination of *δ*^34^S in terms of precision and trueness. In the current study, we did not aim for a high sensitivity. Yet, for analyses of samples with low sulfur contents, substantial improvements may easily be achieved by removing the split in the interface between the EA and the torch of the ICP and directing the entire carrier gas flow of the EA into the plasma. Such an approach is feasible because the EA uses argon instead of helium as the carrier gas, which does not disturb the argon plasma. The use of jet cones might further improve sensitivity, and with both measures combined, samples in the lower nanomole or even picomole range may be measurable with reasonable precision.

## Supporting Information Available

Parameters used for GC, EA, and MC-ICPMS, memory tests, and documentation of R-script for calculation of isotopic ratios (PDF)
R-script for calculation of isotopic ratios (ZIP)
