# Supplementary material for: Continuous-Flow Stable Sulfur Isotope Analysis of Organic and Inorganic Compounds by EA-MC-ICPMS
Source: Anal Chem. 2024 May 13;96(21):8510–7. doi: 10.1021/acs.analchem.4c00439 (PMC11140681; doi:10.1021/acs.analchem.4c00439)
Supplement: Supplementary file 1 — ac4c00439_si_001.pdf [file ac4c00439_si_001.pdf]

Supporting Information

Continuous-flow Stable Sulfur Isotope Analysis of Organic and Inorganic Compounds by EA-MC-ICPMS

Axel Horst[1,2*], Matthias Gehre[1*], Marcus Fahle[2] Steffen Kümmel[1]

[1] Department Technical Biogeochemistry, Helmholtz Centre for Environmental Research – UFZ, Permoserstr. 15, 04318 Leipzig, Germany

[2] Research and Development Centre for Post-Mining Areas, Federal Institute for Geosciences and Natural Resources (BGR), Gaglower Str. 17-18, 03048 Cottbus, Germany

* Corresponding authors: axel.horst@bgr.de, matthias.gehre@ufz.de

number of pages 11

number of figures 1

number of tables 1

Table of Contents

Table S1:	GC, EA, and MC-ICPMS parameters
Figure S1:	Tests for memory effects
SI 1:	Documentation and manual for isotopic ratio calculation using the R-script

Table S1: GC, EA, and MC-ICPMS parameters

Parameter	Applied value
**Elemental analysis**	
Temperature (reactor)	1030°C
Carrier flow (Ar)	80 mL/min
Purge flow (Ar)	70 mL/min
Oxygen flow	15 mL
Separation column	0.8m x 6.35 mm PTFE (Porapak 50-80 mesh)
Oven temperature	70°C
**Gaschromatography**	
Column	60m x 0.32 mm x 1µm (Zebron ZB-1)
Injector	Split/splitless, 250°C
Oven temperature	150°C isothermal
Temperature transferline	280°C
**MC-ICPMS**	
Cooling gas flow (Ar)	14 L/min
Auxiliary gas flow (Ar)	0.75 L/min
Sample gas flow (Ar)	0.86 L/min
Add-gas flow (Ar)	0.5 L/min
RF power	1200 W
Extraction cones	Standard nickel
Extraction voltage	2000 V
Mass resolving power	low (m/Δm ≈300)
Faraday Detectors	L4 (32S), L1 (33S), C (34S)
Amplifiers	L4 (10^{10} Ω), L1 (10^{12} Ω), C (10^{11} Ω)

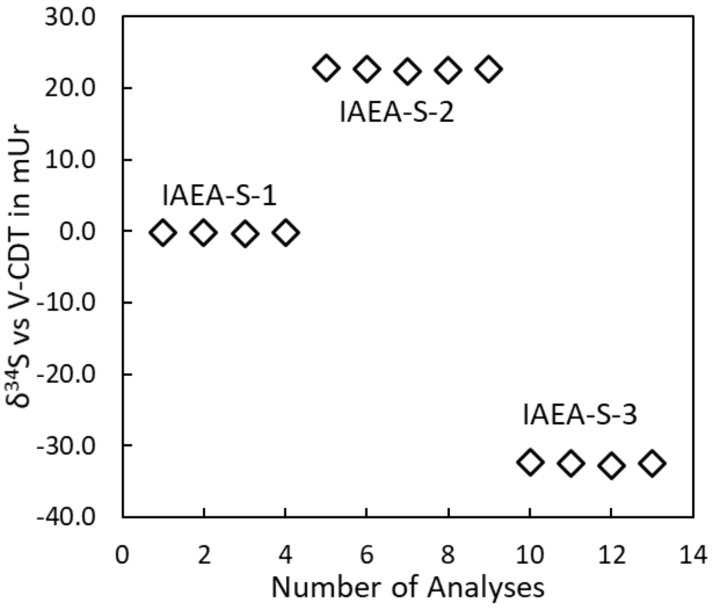

Figure S1. Tests for memory effects. Three IAEA reference materials (Ag_2S) were consecutively analyzed with no other samples or blank combustions interspersed. No memory or carryover was detected.

Supporting Information 1: Documentation and manual for isotopic ratio calculation using the R-script

The Multi Collector Software of the Neptune records the signals transmitted by the Faraday cups. This software was originally developed to calculate isotopic ratios for constant signals, produced by continuous introduction of sample material via a spray chamber, for example. The software is not able to calculate isotopic ratios of transient signals as produced by elemental analysis, for example. To extract isotopic ratios from the data, we wrote a set of scripts in R language.[1,2] These scripts are able to calculate isotopic ratios by two different methods: a) integration of the areas of the transient signals (subsequently called "peaks") as used in the current study and b) regression between two isotopic species as used in previous papers coupling gas chromatography to MC-ICPMS.[3,4] In addition, this script facilitates sensitivity analyses for the different parameters (peak detection etc.) which helps to establish the optimal parameters for each method.

How to use the scripts?

To use the scripts, execute the following steps:

1) Prepare the input text files. For specifications see below.
2) Install *R* software (https://cran.r-project.org/), which is open source. We used version 4.3.1 for our calculations. Additionally, you may install the graphical user interface *RStudio*, of which we used version 2022.07.2.
3) After starting *R* (or *RStudio*) for the first time, install the package *zoo*, which provides functionalities to process time series. Use the command install.packages("zoo").[1] The package only has to be installed once.
4) Start *R* (or *RStudio)* and load the script *Process_isotopic_data.R.*
5) Set the working directory in the script in the according line via *setwd("<your path>")*. Do not use backslashes for the path. An example for the path format is: *"C:/Data/Isotopic_Study/"*
   All input files to be processed have to be in the working directory and need to have "txt" file endings.
6) Specify the calculation parameters in the script (see below), also to define whether a single calculation or a sensitivity study of one parameter will be conducted. Explanations of the parameters are given below.
7) Run the script either by using the command *source("Process_isotopic_data.R")* – note that in this case all changes in the script have to be saved before execution. In *RStudio* you may press CTRL + ALT + R to execute the script or select all lines (e.g. by pressing CTRL + a) and execute them by pressing CTRL + Enter. The script will run through and produce text and graphic files as outcomes of the calculation. An explanation of the generated results is given below.

---

[1] The command also appears in the main script *Process_isotopic_data.R*, but by default the line is not executed. It is marked as a comment by a hashtag (#) at the beginning of the line. Uncomment by removing the hashtag, then the line will be executed together with the script. If you work within a proxy setting, you may have to set the proxy by executing *Sys.setenv(http_proxy="<your proxy>")* and *Sys.setenv(https_proxy="<your proxy>")* first.

Which input data are needed?

Input data files need to be text files with the extension ".txt" that contain tabulated data. The header, which provides additional information, is generally not read – see parameter *lines_to_skip_in_input_file* in the script. Still, the first line of the file has to specify the isotopic species, separated by tabulators. The first element of this line is dismissed (in our case, it is "Trace for Mass:").

The data table below the header has to consist of at least three columns. The first column has to be the time in seconds, the remaining columns the intensities measured for specific isotopic species.

Example file for three isotopic species (as exported from Multi Collector Software Version 3.2):

```
Trace for Mass:     32S  33S  34S
Resolution:    High High High


Time Intensity Intensity Intensity
[sec]     [cps]     [cps]     [cps]


0.000000  2.64E-01  2.48E-03  1.00E-02
1.32E-01  2.62E-01  2.43E-03  9.60E-03
```

Which results are generated?

For each input file, the following result files are generated:

- A text file with the parameters used for calculation of the isotopic ratios
  (file name: <input_file_name>_parameters_used.dat)

- A text file with the calculated isotopic ratios
  (file name: <input_file_name>_results.dat)
  Resulting isotopic ratios derived from integrated areas and from regression are given for each peak in a tabular form. In addition, the last two rows consist of the mean ratios for all peaks and the standard deviation of the ratios of all peaks.

- A text file with the calculated areas under each peak
  (file name: <input_file_name>_areas.dat)
  The file contains the numerically integrated areas calculated for each peak and isotopic species.

- A graphic file visualising the data set
  (file name: <input_file_name>_data.pdf)
  The file contains various pages with plots of the data:
    - A graph of the time series of all isotope species displayed in one plot,

- A graph of the time series of all isotopic species displayed in subplots (one for each isotopic species),
- A single graph for each isotopic species,
- A single graph for each isotopic species, showing only the lowest values (range between 0 and 5 times the lowest measured value in the time series).

In all plots vertical dashed lines indicate the start and end of each identified peak. The graphs can be used to verify whether peaks have been assigned correctly.

- A graphic file visualising the calculated slopes for the data set
(file name: <input_file_name>_slopes.pdf)
The file contains various pages with plots of the calculated slopes, similar to the file <input_file_name>_data.pdf. Instead of plots showing the lowest values in detail, only a plot of the slopes calculated for the isotopic species used to determine the peak starting and end points is given. This detail plot shows the threshold slopes used to define the time window of a peak.

- A graphic file visualising the regression results
(file name: <input_file_name>_regression_isotopic_species.pdf)
Subplots show the isotopic ratios calculated from regression in the title, the two time series of the isotopic species plotted against each other and the coefficient of determination (r^2) in the subtitle for each peak.

In case a sensitivity test of a parameter is conducted, the output files for each parameter value are written to a separate folder. In addition, two summary files are generated:

- Summary file of the results for sensitivity test of a parameter
(file name: <input_file_name>_result_changes_varying_<parameter_varied>.dat)
The table includes the isotopic ratios (both from areal integration and regression) calculated for each peak, isotopic species and parameter value used, plus the mean and standard deviation of the ratios for each parameter value.

- Summary file of the areas for sensitivity test of a parameter
<input_file_name>_area_changes_varying_<parameter_varied>.dat)
The table contains the areas calculated for each peak, isotopic species and parameter value used.

Which parameters can be defined?

The procedure to calculate the isotopic ratios uses a number of parameters. Alterations of the parameters will affect the results of the calculation. Warning: Choosing unsuitable parameter values may cause problems during the execution of the script (e.g. when assigning the starting and ending points of the peaks).

reference_species (by default "32S")
Defines the denominator of the isotopic ratio.

points_considered_to_calculate_slope (by default 11)
For each point in the time series, a slope is calculated. Ascending and descending parts of the peaks are derived based on these slopes. This parameter defines how many points will be taken into account to calculate the slope. For 11 points, 5 points before, 5 points after and the current point are considered.

maximum_gap_in_time_steps_accepted_in_a_slope (by default 25)
Slopes surpassing defined threshold slopes (see below) mark peaks to be investigated. Yet slopes may exceed or fall below these thresholds for a couple of time steps due to noise or fluctuations, while the general trend is still on-going. This parameter is used to avoid starting peaks late or ending them early.

minimum_duration_of_peak_in_secs (by default 30)
Only peaks lasting longer than this minimum duration are used to calculate isotopic ratios.

points_considered_to_calculate_mean_background (by default 10)
Before integrating the area under the peaks of the graphs, the background intensity has to be subtracted. This parameter defines, how many time steps before a peak are considered to calculate the background intensity.

method_to_calculate_background (by default "median")
To calculate the background intensity before a peak starts, select between “median” and “mean”.

isotopic_species_to_derive_peak_window (by default "34S")
If data for more than one isotopic species is available, the time windows of the peaks (i.e. their starting and end points) are only determined for the species defined by this parameter. The time windows of the peaks are then applied to all isotopic species.

threshold_ascending_slope (by default 0.004)
The parts of the graph where its slope exceeds this threshold (i.e. rise beyond normal fluctuation) are classified as ascending conditions (i.e. rising part of a peak). This information is then used to determine the beginning points of the peaks.

threshold_descending_slope (by default -0.004)
The parts of the graph where its (negative) slope falls below this threshold (i.e. fall steeper than normal fluctuations) are classified as descending conditions (i.e. falling part of a peak). This information is then used to determine the end points of the peaks.

name_of_result_folder (by default NA)
The parameter specifies the name of the folder into which all result files are stored. If the parameter is NA (i.e. not available), then all results are written into the current working directory. For sensitivity tests, this parameter is not applied.

lines_to_skip_in_input_file (by default 6)
The number of lines to be skipped in the header of the input file.

Description of the different scripts

File: Process_isotopic_data.R

Running this wrap-up script loads the *zoo* package and the functions contained in the files *Derivation_of_isotopic_ratios.R, Derivation_of_isotopic_ratios_for_one_parameter_set.R, Evaluate_effects_of_parameter_changes.R* and *Load_isotopic_data_from_file.R*.

In the working directory – which can be set within this script -, all text files are identified and assumed to be input files. Specifications of these input files are given above.

Then the list of parameters for calculation of the isotopic ratios, called *parameters_used*, is set. The calculation is done for all text (i.e. input) files with the set parameters. There is the possibility to vary one of the parameters, while keeping the others constant (i.e. to conduct a sensitivity analysis). To vary one parameter, define the argument *parameter_to_vary* in the call of the function *Derivation_of_isotopic_ratios*. Use the parameter names specified in the list *parameters_used*. Define the parameter values to be tested in the argument *parameter_to_vary_values*. When not defining *parameter_to_vary*, the script calculates the isotopic ratios only for *parameters_used*; that is, only one calculation is done.

File: Load_isotopic_data_from_file.R

Contains the function *Load_isotopic_data_from_file*, which loads the measured data from the input text file. The only arguments of the function are the name of the file (*file_name*) and the number of lines to be skipped in the header of the input file (*lines_to_skip_in_input_file*).

After skipping the defined number of lines, the tabulated data is read and stored as a variable named *isotopic_data*. Afterwards the names of the isotopic species are read from the first line. The first of the elements in this line, which have to be separated by tabulators, will be discarded. In our case, it is "Trace for Mass:" The remaining elements are stored as the variable *isotopic_species*. The variable is used to rename the columns of the data frame *isotopic_data*, which is then converted into the time series format of the package *zoo*.

The function returns the *zoo* time series *isotopic_data*, the *isotopic_species* variable and a string *file_stem*, which is the name of the input file apart from the file ending.

The function is able to load data with any number of columns containing isotopic data.

File: Derivation_of_isotopic_ratios.R

Contains the function *Derivation_of_isotopic_ratios* that loads isotopic data from a specified file (specified in the function argument *file_name*), using the function *Load_isotopic_data_from_file*.

In case only one parameter set is given (i.e. the argument *parameter_to_vary* was not set), the function *Derivation_of_isotopic_ratios_for_one_parameter_set* is called with the given parameter set.

If one parameter is varied; that is, a sensitivity test is done, the isotopic ratios are calculated within a loop for the different parameter sets. In case, the parameter *threshold_ascending_slope* is varied, the parameter *threshold_descending_slope* is altered as well and set to the opposite of *threshold_ascending_slope*. Result files are saved to a sub-directory, whose name consists of the word results, the parameter varied and the parameter value. For example, the directory

“Results_threshold_ascending_slope_0.004” includes all result files for the parameter set, in which the *threshold_ascending_slope* was set to 0.004. The parameter *threshold_descending_slope* was set to -0.004 (see above).

File: Derivation_of_isotopic_ratios_for_one_parameter_set.R

Contains the core function to derive the isotopic ratios, called *Derivation_of_isotopic_ratios_for_one_parameter_set*. The function writes all result files into the working directory, unless the argument is *name_of_result_folder* defined. The following steps are conducted to calculate the isotopic ratios:

1. Determine the peak time windows
   First, the script calculates the slopes at all points of the time series for each isotopic species. Then points belonging to ascending parts of the peaks are identified as those where slopes exceed *threshold_ascending_slope* (i.e. the rising slope is steeper (more positive) than normal fluctuations). Then all points belonging to descending parts of the peaks are identified. Here slopes are lower than *threshold_descending_slope* (i.e. the falling slope is steeper (more negative) than normal fluctuations).
   Knowing all time steps with descending slopes, lagged differences between these time steps are calculated. This determines the time between those points having descending slopes. The end of a single peak is reached, when the distance to the next time step with descending slope exceeds the *maximum_gap_in_time_steps_accepted_in_a_slope* (i.e. descending slopes are absent for a certain time). The latter parameter is needed, as the (negative) slope may exceed *threshold_descending_slope* for several consecutive time steps, even though the signal is still decreasing (e.g. tail of the peak). The end of the very last peak is then added as the last time step with descending conditions. The starting point of each peak is determined in a similar manner. Here, the very first time step associated with an ascending slope has to be added as the start of the very first peak. After all potential start and end points of peaks are identified, some data is cleared out (e.g. when the first ending is before the first starting point, if there is multiple starting points with no assigned ending in between, etc.). Finally, all peaks shorter than *minimum_duration_of_peak_in_secs* (i.e. artefacts) are erased.

2. Calculate isotopic ratios by integration
   First, the background level is calculated for a number of time steps preceding the peak (parameter *points_considered_to_calculate_mean_background*). The parameter *method_to_calculate_background* defines whether mean or median is used. Then the peak area is calculated by trapezoidal approximation. The mean background is subtracted beforehand. The isotopic ratio of two chosen isotopes is then determined by dividing the respective areas of the mass traces.

3. Calculate isotopic ratios by regression
   The measured intensities for two isotopic species belonging to a chromatographic peak signal are plotted against each other. The calculated slope represents the isotopic ratio.

Afterwards, result and parameter tables are generated and plots are created.

File: Evaluate_effects_of_parameter_changes.R

Contains the function *Evaluate_effects_of_parameter_changes* that generates summary files for sensitivity analysis. The function has three arguments highlighted in *italics* in the following. It searches the *directory_where_results_are_saved* for folders containing result files (using *pattern_to_recognize_result_folder*), then loads the value of the *parameter_altered* and all results. Summaries are then generated, but only when the number of peaks identified is the same for all parameter settings.

References:

(1) R Core Team. R: A Language and Environment for Statistical Computing. R Foundation for Statistical Computing, Vienna, Austria. URL Http://Www.R-Project.Org/; 2013.

(2) Zeileis, A.; Grothendieck, G. Zoo: S3 Infrastructure for Regular and Irregular Time Series. J. Stat. Softw. **2005**, 14, 1–27.

(3) Horst, A.; Renpenning, J.; Richnow, H.-H.; Gehre, M. Compound Specific Stable Chlorine Isotopic Analysis of Volatile Aliphatic Compounds Using Gas Chromatography Hyphenated with Multiple Collector Inductively Coupled Plasma Mass Spectrometry. Anal. Chem. **2017**, 89 (17), 9131–9138.

(4) Kümmel, S.; Horst, A.; Gelman, F.; Strauss, H.; Richnow, H. H.; Gehre, M. Simultaneous Compound-Specific Analysis of δ33S and δ34S in Organic Compounds by GC-MC-ICPMS Using Medium- and Low-Mass-Resolution Modes. Anal. Chem. **2020**, 92 (21), 14685–14692.
